# Effect of Cold and Warm Rolling on the Particle Distribution and Tensile Properties of Heterogeneous Structured AlN/Al Nanocomposites

**DOI:** 10.3390/ma13184001

**Published:** 2020-09-09

**Authors:** Lei Song, Fenghua Lu, Feng Jin, Jinfeng Nie, Guiliang Liu, Yonghao Zhao

**Affiliations:** 1 Nano and Heterogeneous Materials Center, Nanjing University of Science and Technology, Nanjing 210094, China; 13646114130@163.com (L.S.); lufenghua12345@163.com (F.L.); jf19990613@163.com (F.J.); 2 Key Laboratory for Liquid-Solid Structural Evolution and Processing of Materials, Ministry of Education, Shandong University, Jinan 250061, China; 18678391822@163.com

**Keywords:** aluminum matrix composites, particle distribution, heterogeneous structure, tensile strength, rolling, AlN

## Abstract

Recently, heterogeneous structured metals have attracted extensive interest due to their exciting mechanical properties. In this work, an AlN/Al nanocomposite with heterogeneous distribution of AlN nanoparticles was successfully prepared by a liquid-solid reaction method combined with subsequent extrusion deformation, which behaves an excellent synergy of tensile strength and ductility. In order to further reveal the particle distribution evolution and the tensile property response during further deformation, a series of rolling treatments with different thickness reductions under room temperature and 300 °C was carried out. The results show that the yield strength and tensile strength of the composites increase significantly from 238 MPa, 312 MPa to 312 MPa, 360 MPa after 85% rolling reduction at 300 °C. While the elongation decreased from 15.5% to 9.8%. It is also noticed that the elongation and tensile strength of the nanocomposites increases simultaneously with increasing deformation. It is considered that the aluminum matrix strengthening effect accounts for the strength enhancement. The AlN spatial distribution in the matrix becomes more homogeneous gradually during the rolling, which is supposed to reduce the stress concentration between the particle and matrix and then promote the ductility increase.

## 1. Introduction

As an important part of metal matrix composites, aluminum matrix composites (AMCs) provide a combination of low density, high strength, excellent wear resistance and corrosion resistance for automotive industries and aerospace applications [1,2,3,4]. Particularly, aluminum matrix composites with different microstructural design by in situ fabrication such as melting methods and spark plasma sintering (SPS) are popular owing to their advantages [5,6,7,8]. However, the purpose is always to obtain a uniform distribution of reinforcement regardless of which processing method. In fact, the situation has been confirmed by many investigations [9,10,11] that the homogenous microstructure can only play a limited role for the mechanical properties.

Recently, AlN/Al nanocomposites reinforced with inhomogeneous distributed AlN network have been prepared by liquid-solid in situ reaction in our previous work [12]. It is revealed that the network-rich region can be effectively strengthened by AlN network, and the network-lean region supplies the ductility of composites due to the coarse matrix grains. This strategy is similar to the theory proposed by Lu that the comprehensive performance of composites can be improved by assembling metals with other components to form novel reinforcements or hierarchical structures [13]. Generally, further deformation is always needed to optimize the mechanical properties of composites during the manufacturing process of a specific part. Therefore, it is necessary to study the effect of subsequent deformation process on the microstructure and the mechanical properties. In order to further reveal the particle distribution evolution and tensile property response of the heterogeneous structured AlN/Al nanocomposites, a series of rolling deformations with different thickness reductions under room temperature and 300 °C was carried out. In this work, the particle distribution has been changed during the rolling processes at different temperatures and with different strains. And then the mechanical responses are analyzed based on the microstructure evolution and strengthening mechanism, which can also provide guidance for the plastic forming for other metal matrix composites.

## 2. Materials and Methods

The commercial pure Al powders (99.7%, all compositions quoted in this work are nominal values in mass fractions unless otherwise stated, with an average diameter of ~20 μm) and hexagonal boron nitride powders (99%, with an average particle size of ~2 μm) were used as raw materials supplied by Shandong Al&Mg Melt Technology Co. Ltd. (Jinan, China), and the initial AlN/Al composite was fabricated according to the method reported in previous work [12]. The mass fraction of AlN was 8.2 wt.% and a extrusion ratio of 7:1 was selected. Furthermore, some plates with 30 mm in length, 20 mm in width, and 10 mm in thickness were cut from the initial nanocomposites for further room temperature (RT) rolling and warm rolling at 300 °C, respectively. The experiment of rolling was performed by laboratory rolling mill and the reduction in thickness was about 0.5 mm per rolling pass, meanwhile, the nanocomposite was preheated for 10 min before each warm rolling pass. The rolled thickness reductions were 16%, 40% and 85% after several passes.

The particle distribution and grain structure of the nanocomposites were ascertained with a scanning electron microscope (FESEM) (FEI, Hillsboro, OR, America) equipped with an Oxford energy dispersive X-ray spectrometer (EDS) (FEI, Hillsboro, OR, America) and electron backscattered diffraction (EBSD) (Zeiss, Jena, Thuringia, Germany) and a transmission electron microscope (TEM) (FEI, Hillsboro, d America). The samples for microstructure observation were etched using 0.5 vol.% hydrofluoric acid after mechanical polishing in order to observe the AlN clearly. The nanocomposites for EBSD characterization were mechanically polished and then electrochemically polished in a mixed solution with the composition of 10% perchloric acid and 90% ethanol at 20 V for 15 s. All EBSD data were analyzed using the Channel 5 software. The Vickers hardness experiment was carried out for ten times for each nanocomposite on the surface of rolling direction (RD)-normal direction (ND) plane using HMV-G 21DT at the load of 0.98N and holding for 15 s. The tensile tests were carried out at room temperature for five times for every nanocomposite using Walter+bai LFM 20 kN universal testing machine at a constant crosshead speed of 1.8 mm/min. The gauge size of the specimens was 10 mm in length and 2.5 mm in width and the loading direction was parallel to RD of the sheets.

## 3. Results

### 3.1. Microstructure and Tensile Properties of AlN/Al Composites at RT Rolling

**Figure 1:**
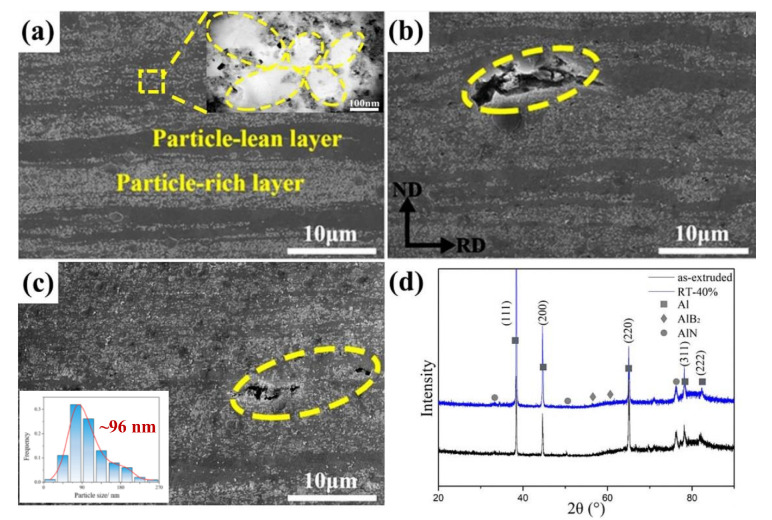
Microstructures and phase identification of AlN/Al composites with different rolling reductions at room temperature: (**a**) as-received sample; (**b**) 16%; (**c**) 40%, the inset showing the size distribution of AlN particles; (**d**) X-ray diffraction (XRD) patterns of as-received sample and 40%.

Figure 1 shows the microstructure and phase identification of AlN/Al composites before and after RT rolling. The received as-extruded sample with heterogeneous particle distribution can be seen in Figure 1a, which contains a particle-rich layer (PRL) and a particle-lean layer (PLL). It is known that the in situ formed nanoparticles are prone to agglomerate and form particle clusters in the matrix, which are distributed along the grain boundaries during the solidification process as revealed in our previous study [14]. Thus, particle-rich zones and particle-free zones were formed in the microstructure. The particle-free zones were elongated during the following extrusion and rolling deformation process, and then the PLL formed as seen in Figure 1a. At a higher magnification, the nano-AlN network distributed in PRL can be seen clearly in the TEM image. When the rolling thickness reduction at room temperature reaches 16%, the distribution of particles tends to be uniform and the space between the PRL and PLL was reduced as displayed in Figure 1b. However, a crack of about 10 μm was found, which indicates the poor deformation ability of AlN/Al composite at room temperature. It is supposed that severe strain localization occurred in the particle-rich zones and led to the stress concentration during rolling deformation, which reduced the deformation ability of the composites. Then, the micron defects initiated in the strain localization areas and propagated rapidly during the subsequent tensile deformation, which reduced the ductility dramatically. With the further increase of thickness reduction to 40%, the particle distribution was much more dispersive. It was also noticed that there were many cracks on the composite surface when the thickness reduction reached 78%, and then the microstructure was not provided without further investigation. Besides, the particle distribution of AlN in the sample rolled with 40% reduction was also calculated as shown in the inset of Figure 1c, and the mean size of AlN particles in the matrix was about 96 nm as shown by the inset of Figure 1c. Figure 1d shows XRD patterns of the as-received sample and the sample rolled with 40% reduction at RT, which reveals that AlN phase formed in the matrix, and a small amount of AlB_2_ also formed. It can be seen that no phase transition occurred during the rolling deformation at RT.

**Figure 2:**
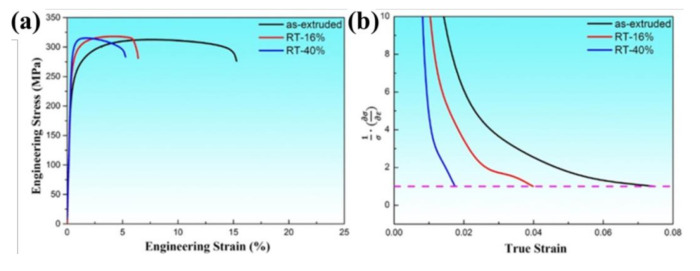
(**a**) Engineering tensile stress and strain curves of AlN/Al nanocomposites with different rolling reductions at room temperature (RT); (**b**) the obtained strain hardening rates of the nanocomposites before and after rolling.

To investigate the influence of room temperature rolling on the tensile properties of the AlN/Al nanocomposites, Figure 2a exhibits the engineering stress and strain curves with different rolling reductions at RT and the detailed tensile property data are shown in Table 1. It can be seen that the yield strength (YS) of composite increases from 238 MPa to 276 MPa after 16% thickness reduction, and when the reduction further increases to 40%, YS reaches to 300 MPa. As is widely known, the accumulation of dislocation and grain refinement during rolling deformation are the main factors for the YS increase during the rolling deformation. Meanwhile, the elongation of composite after rolling significantly decreased from 15% to 6.2% and 5% due to the reduced dislocation storage ability of the matrix α-Al grains, respectively. It is also worth noting that the ultimate tensile strength (UTS) of the samples has been hardly changed in spite of the rise of YS. According to this phenomenon, it can be explained by Figure 2b that the strain hardening rate decreases with increasing reduction, which accounts for the dramatic loss of ductility after rolling deformation at RT. Therefore, it shows that rolling deformation at RT can only increase the YS of the composites. However, the comprehensive mechanical properties including the UTS and elongation have not been improved as a result of the reduced strain hardening rate and micron crack defects.

### 3.2. Microstructure and Tensile Properties of AlN/Al Composites after 300 °C Rolling

**Figure 3:**
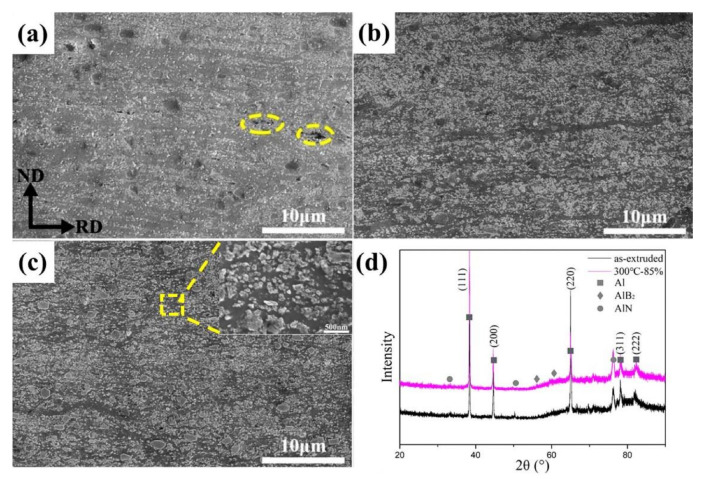
Microstructures and phase identification of AlN/Al nanocomposites with different rolling reductions at 300 °C: (**a**) 16%; (**b**) 40%; (**c**) 85%; (**d**) XRD pattern of the as-received sample and 85%.

In order to further study the effects of the warm rolling deformation on the microstructure and mechanical properties of AlN/Al composites, the AlN/Al composite was subjected to 300 °C warm rolling deformation with thickness reduction of 16%, 40% and 85%, respectively. The microstructure of AlN/Al composites after 300 °C rolling is shown in Figure 3. The inhomogeneous distribution of AlN particles became much more uniform after 40% reduction, as shown in Figure 3b. It is also noticeable that a few micron cracks with a size smaller than 3 μm can be seen in the sample with 16% reduction as shown in Figure 3a, while the crack size can hardly be observed with increasing rolling reduction. It is apparent that the deformation ability or plasticity is much better at a relatively high temperature and the strain localization and stress concentration were inhibited during the elevated temperature rolling deformation. When the rolling thickness reduction increased to 85%, the spatial distribution of AlN particles was much more homogeneous as shown in Figure 3c. Figure 3d shows the XRD patterns of the as-received sample and the sample rolled with 85% reduction at 300 °C, which also reveals that no phase transition occurred during the warm rolling deformation.

**Figure 4:**
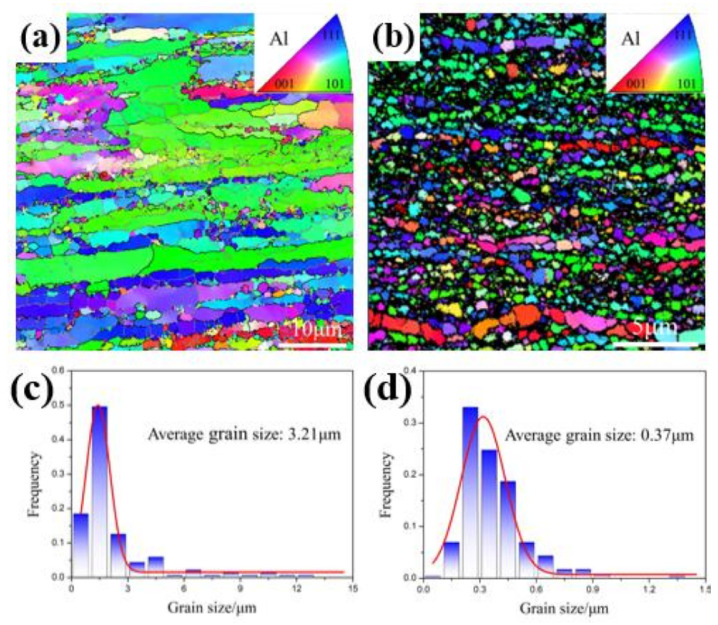
Electron backscattered diffraction (EBSD) characterization of the AlN/Al nanocomposites along the longitudinal direction before and after rolling treatment: (**a**,**c**) inverse pole figure (IPF) map of matrix grains of the as-extruded composites and the size distribution of matrix grains; (**b**,**d**) IPF map of matrix grains of the composite after 85% rolling at 300 °C and the size distribution of matrix grains.

The matrix grain structure after rolling treatment was also analyzed by EBSD images. Figure 4a–d shows the inverse pole figure (IPF) maps and grain size statistic distribution of α-Al grains in the extruded composites and that after 85% rolling reduction at 300 °C. As displayed in Figure 4a, a long elongated grain structure with a large width range of submicron to over 5 μm can be seen in the as-received sample. It is reasonable to believe that the heterogeneous distribution of AlN particles and the particle-lean layers in the matrix leads to the elongated grain structure, which agrees well with the SEM image (Figure 1a). In addition, there are a few recrystallized grains with equiaxed morphologies. The matrix grain size distribution is depicted in Figure 4c and the average gain size was ~3.21 μm. Figure 4b shows the grain structures of the sample after 85% rolling reduction, which indicates that the coarse grain structure had been refined significantly and had a finer average size of ~0.37 μm (Figure 4d) compared with the extruded one. Based on the above results, this indicates that most of the coarse grains were refined significantly to an ultra-fine-grained (UFG) structure due to the occurrence of dynamic recrystallization accompanied with homogenous dispersion of AlN during the rolling deformation.

**Figure 5:**
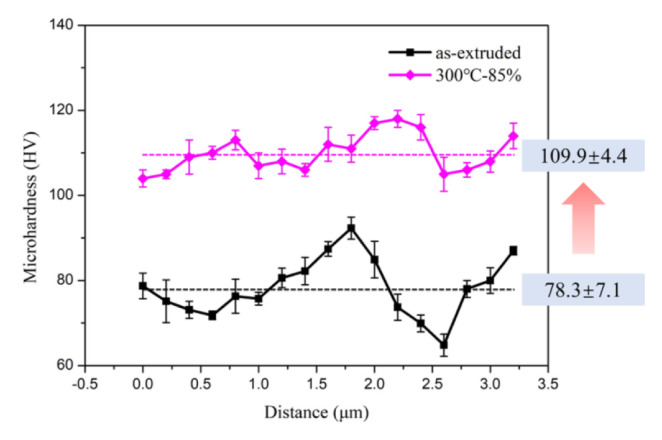
Microhardness (HV) of AlN/Al composites before and after rolling for 85% thickness reduction at 300 °C.

Figure 5 shows the Vickers hardness distribution maps of AlN/Al composites before and after rolling for 85% thickness reduction at 300 °C. It can be clearly observed that the hardness distribution of composites before rolling was inhomogeneous due to the heterogeneous distribution of AlN, as the high value was about 85 HV and the low value was about 65 HV. Combined with the microstructures shown in Figure 1a, it can be seen that the particle-rich layers with more AlN had a higher hardness, while the particle-lean zones had a lower hardness. The heterogeneous distribution of reinforcement particles led to an inhomogeneous distribution of hardness. After 85% rolling thickness reduction, the hardness of the composites was increased significantly and the hardness distribution also became more uniform with an average value of ~110 HV. It is apparent that the increased dislocation density by the obstruction of reinforcement resulted in the hardness enhancement [15]. Meanwhile, the change of spatial distribution and more homogenous AlN distribution after rolling led to the uniformity of hardness, which is consistent with the microstructure in Figure 3c.

**Figure 6:**
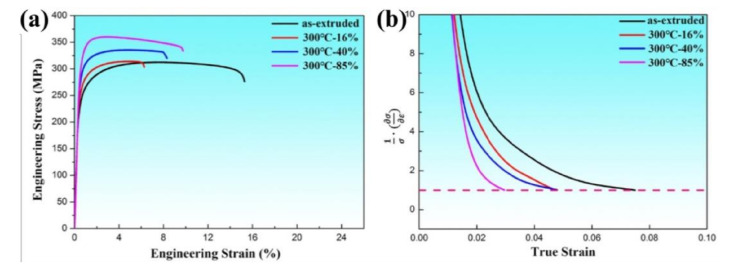
(**a**) Engineering tensile stress and strain curves of AlN/Al nanocomposites with various rolling reductions at 300 °C; (**b**) the strain hardening rate curves of the four nanocomposites.

Furthermore, the engineering stress and strain curves of AlN/Al composites with different rolling thickness reductions from 16% to 85% at 300 °C are shown in Figure 6a and the detailed data are shown in Table 2. It can be seen that with the increasing rolling reduction, the yield strength of composites increased from 238 MPa to 255 MPa, 286 MPa and 312 MPa. The variation rule is similar to the above results at RT rolling. It is also noticeable that the YS of the 300 °C rolling samples was lower than of the sample after RT rolling with the same deformation reduction. The main reason is that dynamic recovery happened during warm rolling deformation and the total storage of dislocations in the matrix grains introduced by plastic deformation reduced to a certain extent [16]. However, it can also be seen that UTS of composites continuously increased from 312 MPa to 360 MPa after 85% thickness reduction. As is shown in Figure 6b, the corresponding strain hardening also decreased with the increasing thickness reduction owing to the limited storage capacity of dislocations in refined aluminum matrix grains, as shown in Figure 4b [17], but it is still higher than that under the same reduction at room temperature, owing to the dynamic recovery and recrystallization occurred in the matrix grains during 300 °C rolling deformation. At the same time, the elongation of composites after 16% thickness reduction was also reduced significantly owing to reduced working hardening rate of ultra-fine-grained Al matrix. Furthermore, it can be seen that, with increasing rolling reduction, the elongation of composite increased continuously from the initial 6.3% to 8.3%, 9.8%, respectively. This shows that the tensile strength and elongation of the nanocomposites were improved simultaneously with the increasing strain deformation. As indicated by Figure 6b, the strain hardening was reduced, as shown in Figure 2b, indicating the reduced dislocation ability of the matrix grains. Some researchers [18,19] indicate that the reinforcement disperses more uniformly during rolling deformation and that it will then inhibit the strain location and stress concentration, which is beneficial to the total elongation increase to a certain extent. However, the total elongation was still smaller than that of the as-received one, due to the significantly reduced dislocation storage capacity of the UFG matrix structure in the rolled nanocomposites.

## 4. Discussion

Generally, the particle distribution in the composites plays a key role in determining the mechanical properties of the composites. The tensile strength and ductility variation during the rolling deformation can be rationalized from the particle spatial distribution change. It has been shown that in the case of the same grain size, the composites with heterogeneous network architecture [20,21,22] show higher strength than those with homogeneous particle distribution. Meanwhile, it is also evident that, for different composites with network structure and homogeneous distribution, the strengthening effect contributed from the load-bearing capability is distinct. The particle network structure has a higher load-bearing efficiency than that of an isolated reinforcement particle. Roy [23] used the stress concentration factor to describe the inhomogeneous distribution of stress in matrix and reinforcement particles. Both the experiment [23] measured by energy dispersive X-ray diffraction and finite element method (FEM) simulation [24,25,26] indicate that the stress concentration factor of reinforcement increases in plastic stage and falls in failure stage. Meanwhile, the factor of composites with network structure is larger than that with homogeneous microstructure; it can transfer more load from the matrix to reinforcement particles. Therefore, the high load-bearing capability of network architecture contributes to the higher strengthening effect. However, the research also suggests that strain localization induced by high load-bearing capability causes the crack initiation and the crack propagates rapidly through the network, leading to the decrease of total elongation.

It is noticeable that the AlN/Al nanocomposite with heterogeneous distributed AlN network has a superior elongation of 15% than that of the relatively homogenous distributed one (Figure 5). The main reason is that the heterogeneous lamella structure forms in Al matrix discussed in our previous work [27]. The coarse elongated matrix grains in the particle-lean zones can supply larger space for dislocation accumulation and sliding during the plastic deformation, which is a key factor for the large ductility of the original sample for the heterogeneous structured composites. Therefore, the dislocation storage ability of the aluminum matrix containing coarse elongated grains plays a crucial role in the ductility enhancement of the composites. A further study such as a short time annealing is need to repair the dislocation storage capacity to enhance the ductility, obtaining a better strength and ductility synergy.

**Figure 7:**
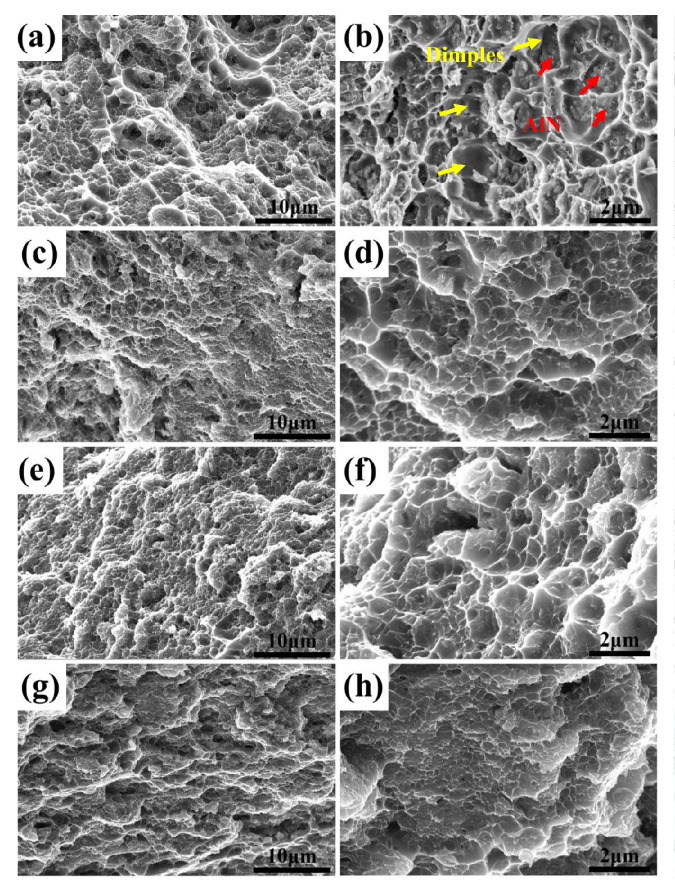
The fracture surfaces of AlN/Al composites after different rolling thickness reductions at 300 °C: (**a**,**b**) 0%; (**c**,**d**) 16%; (**e**,**f**) 40%; (**g**,**h**) 85%.

Furthermore, the fracture surfaces of samples with different rolling reductions at 300 °C are shown in Figure 7. A large number of dimples indicated by the yellow arrows can be found for all samples, which indicates that a typical ductile facture happens in these AlN/Al composites with different particle distribution and AlN can be seen on the surface as indicated by the red arrows. As is widely known, the ductile damage mechanism consists of void nucleation and interface decohesion. It is considered that most of the voids nucleated at the AlN/matrix interfaces and AlN nanoparticles can be seen in the center of dimples as shown in Figure 7b. For the composites before and after rolling deformation, the void nucleation and propagation are the main fracture style, similar to previous study [28]. After rolling deformation, the original dimples in different sizes have become more homogeneous, which is consistent with the change of AlN distribution (Figure 3). Furthermore, the morphology of dimples also becomes flatter due to uniform distribution after rolling deformation, which indicates the enhanced ductility of the composites with increasing deformation to a certain extent. In a summary, the strength of the AlN/Al nanocomposites has been significantly improved with a slight loss of ductility after warm rolling deformation. It is thought that the tensile strength and ductility combination will be optimized by a subsequent annealing treatment in our further study.

## 5. Conclusions

1. The AlN/Al nanocomposite with heterogeneous distributed AlN was successfully prepared by a liquid-solid reaction method combined with extrusion. The effect of rolling deformation at RT and 300 °C on the particle distribution and tensile property response were studied in detail. It shows that the RT rolling deformation can effectively increase the yield strength of composites; however, the elongation drops significantly owing to the reduced strain hardening rates and the formed micron cracks.
2. The mechanical properties of network AlN/Al composites are significantly improved via warm rolling at 300 °C. The YS and UTS of the nanocomposites increase from 238 MPa, 312 MPa to 312 MPa, 360 MPa after 85% rolling reduction. It is supposed that the increased strengthening from grain boundary strengthening of the UFG aluminum matrix accounts for the strength enhancement.
3. The heterogeneous network architecture of AlN particles is gradually changed during the rolling deformation, and the more homogeneous AlN distribution is obtained with increasing strain. The strength and ductility are simultaneously increased during the warm rolling and a good combination of tensile strength (360 MPa) and ductility (9.8%) is obtained for a pure aluminum matrix based composites. The more homogenous distribution of AlN particles can reduce the stress concentration and is the main reason for the increased elongation.

## Figures and Tables

**Table 1:** Tensile properties of AlN/Al composites with different rolling reductions at RT.

Samples	YS (MPa)	UTS (MPa)	Elongation (%)
As-extruded	238	312	15
RT-16%	276	317	6.2
RT-40%	300	315	5

**Table 2:** Tensile properties of AlN/Al nanocomposites after different rolling reductions at 300 °C.

Samples	YS(MPa)	UTS(MPa)	Elongation (%)
As-extruded	238	312	15.5
300 °C-16%	255	314	6.3
300 °C-40%	286	335	8.3
300 °C-85%	312	360	9.8
